# Evaluation of Intervention Acceptability in Community Studies With Individuals and Dyads Affected by Dementia

**DOI:** 10.1093/geroni/igaa057.528

**Published:** 2020-12-16

**Authors:** Melissa Harris, Marita Titler

**Affiliations:** 1 University of Michigan, Madison Heights, Michigan, United States; 2 University of Michigan, Ann Arbor, Michigan, United States

## Abstract

Multiple non-pharmacologic interventions for symptom management in community dwelling individuals with dementia have demonstrated effectiveness, but have had limited uptake in practice. Prior reviews have evaluated acceptability of interventions for caregivers, but none have evaluated interventions for care recipients with dementia and dyads. This review synthesized the evidence about intervention acceptability for dyads (individuals with dementia and informal caregivers) and individuals with dementia residing at home. Four databases were searched (PubMed, CINAHL, AgeLine, PsycINFO) using inclusion criteria of: intervention studies, community dwelling individuals with dementia or dyads of care recipients and informal caregivers, non-pharmacologic intervention, evaluation of intervention acceptability. Gray literature and non-English articles were excluded. 173 citations were screened by title and abstract, 38 were reviewed by full text, and 19 studies were included. 18 studies focused on dyads, and 13 different non-pharmacologic intervention types were evaluated across studies. Qualitative (n=3), quantitative (n=8) and mixed methods (n=8) were used to evaluate acceptability. Approaches and measures of acceptability included field notes, behavioral checklists, focus groups, semi-structured interviews, questionnaires, and completion rates of intervention sessions and outcome measures. Although participants’ benefit and satisfaction with the interventions were high across studies, variability in definitions of acceptability, the methods and measures used constrain the interpretation and generalizability of findings. Psychometric properties of quantitative questionnaires were not addressed even as the most basic level of face or content validity. To enhance the applicability of non-pharmacologic treatments for this population, future research should emphasize the evaluation of intervention acceptability, as well as effectiveness.

